# Characterization of Mammary Tumors Arising from MMTV-PyVT Transgenic Mice

**DOI:** 10.3390/cimb45060286

**Published:** 2023-05-24

**Authors:** Chien-Liang Liu, Wen-Chien Huang, Shih-Ping Cheng, Ming-Jen Chen, Chi-Hsin Lin, Shao-Chiang Chang, Yuan-Ching Chang

**Affiliations:** 1 Department of Surgery, MacKay Memorial Hospital and Mackay Medical College, Taipei 104217, Taiwan; chess@mmh.org.tw (C.-L.L.); wjhuang0.4909@mmh.org.tw (W.-C.H.); disgras@mmh.org.tw (S.-P.C.); mjchen@mmh.org.tw (M.-J.C.); 2 Institute of Biomedical Sciences, Mackay Medical College, New Taipei City 252005, Taiwan; 3 Department of Medical Research, MacKay Memorial Hospital, Taipei 104217, Taiwan; lcs2174.b519@mmh.org.tw (C.-H.L.); shao.f109@mmh.org.tw (S.-C.C.); 4 Department of Bioscience Technology, Chung Yuan Christian University, Taoyuan City 320314, Taiwan

**Keywords:** breast cancer, MMTV-PyVT mice, whole-exome sequencing

## Abstract

Among genetically engineered mouse models of breast cancer, MMTV-PyVT is a mouse strain in which the oncogenic polyoma virus middle T antigen is driven by the mouse mammary tumor virus promoter. The aim of the present study was to perform morphologic and genetic analyses of mammary tumors arising from MMTV-PyVT mice. To this end, mammary tumors were obtained at 6, 9, 12, and 16 weeks of age for histology and whole-mount analyses. We conducted whole-exome sequencing to identify constitutional and tumor-specific mutations, and genetic variants were identified using the GRCm38/mm10 mouse reference genome. Using hematoxylin and eosin analysis and whole-mount carmine alum staining, we demonstrated the progressive proliferation and invasion of mammary tumors. Frameshift insertions/deletions (indels) were noted in the Muc4. Mammary tumors showed small indels and nonsynonymous single-nucleotide variants but no somatic structural alterations or copy number variations. In summary, we validated MMTV-PyVT transgenic mice as a multistage model for mammary carcinoma development and progression. Our characterization may be used as a reference for guidance in future research.

## 1. Introduction

Genetically engineered mouse models have contributed extensively to our understanding of disease processes. To study tumorigenesis in breast cancer, mammary-specific or -selective promoters are commonly used in transgenic mouse models. The most widely used regulatory element for inducing mammary-selective transgene expression is the mouse mammary tumor virus (MMTV) long terminal repeat and the promoter of the whey acidic protein (*Wap*), which encodes the milk serum protein [1]. Various strategies involving the loss of tumor suppressor genes or gain of function in oncogenes such as *Erbb2*, *Myc*, *Ccnd1*, polyoma virus middle T (PyVT), and *Hras* have been used to investigate the initiation and progression of breast cancer. Polyoma virus, such as SV40, is a DNA tumor virus that contains a potent transforming protein, and it was demonstrated that the middle T antigen is required for transformation [2]. The advantages of MMTV-PyVT transgenic mice are their development of synchronous multifocal tumors in all of the mammary glands with a short latency, as well as a high prevalence of pulmonary metastasis [3]. As such, MMTV-PyVT is the most commonly used genetically engineered mouse model for cancer research and serves as a preclinical platform for therapeutic testing [4].

Although MMTV-PyVT transgenic mice are frequently used in preclinical research, few studies have performed morphologic and genetic analyses of mammary tumors arising from MMTV-PyVT mice. Comprehensive genomic profiling revealed that MMTV-PyVT tumors showed luminal-like gene expression patterns [5,6]. Another study evaluated the phenotypes of multiple immunohistochemical markers and demonstrated that Ki-67 expression progressively increased during tumor progression [7]. In the current study, we examined histological characteristics, including whole-mount carmine alum staining, and determined genomic alterations using whole-exome sequencing (WES). The results of this study may shed light on the pathogenesis of the development of breast neoplasms in this model.

## 2. Materials and Methods

### 2.1. Transgenic Mice

This study was conducted in accordance with the Guidelines for the Care and Use of Laboratory Animals published by the Council of Agriculture of Taiwan and was approved by the Institutional Animal Care and Use Committee of MacKay Memorial Hospital (MMH-A-S-108-22). B6.FVB-Tg(MMTV-PyVT)634Mul/LellJ transgenic mice from the Jackson Laboratory (Bar Harbor, ME, USA) represent a mouse strain in which an oncogene derived from the polyoma virus is expressed in the mammary gland tissues, driven by the mammary tumor virus promoter [8]. To maintain a live colony, hemizygous male B6.FVB-Tg(MMTV-PyVT)634Mul/LellJ mice were bred with C57BL/6J inbred females (purchased from BioLASCO, Taipei, Taiwan). Dr. Ming-Shen Dai (Tri-Service General Hospital, Taipei, Taiwan) generously provided the MMTV-PyVT mice [9].

### 2.2. Spontaneous Mammary Tumor

Hemizygous MMTV-PyVT mice develop spontaneous mammary tumors that closely resemble the progression of human breast cancer from premalignant to malignant breast disease. Both the body weight and tumor volume of each mouse were monitored twice a week for the duration of the study. Tumor volume was calculated using the modified ellipsoidal formula as length × width^2^ × 1/2 [10]. Mammary glands and mammary tumors were collected from at least five mice per time point for histological analysis. Normal mammary gland tissue samples were obtained from age-matched C57BL/6J female mice.

### 2.3. Histology and Whole-Mount Analysis

After sacrifice of the mouse, thoracic (second and third) and abdominal (fourth) mammary glands and tumors were harvested and fixed in 10% formalin at room temperature overnight. The tissues were paraffin-embedded, sectioned, and stained as per the standard hematoxylin and eosin (H&E) procedures. To prepare the whole mounts, the mammary glands were transferred onto a positively charged microscope slide (Muto Pure Chemicals, Tokyo, Japan). The mammary glands were spread out as much as possible without tearing the tissue and were fixed with Carnoy’s solution composed of 60% ethanol, 30% chloroform, and 10% glacial acetic acid for 24 h. The glands were then stained with carmine alum (Sigma-Aldrich, St. Louis, MO, USA) for 48 h, de-stained in 70% ethanol with 2 mM HCl for 4 h to remove excessive dye, and dehydrated in graded alcohol solution [11]. The dehydrated tissue slides were submerged in xylene for at least 12 h for adipose tissue clearing.

### 2.4. Whole-Exome Sequencing

DNA from mammary tumors and matched tails were extracted from two MMTV-PyVT mice at 12 weeks of age using the DNeasy Blood and Tissue Kits (Qiagen, Hilden, Germany). The extracted DNA was treated with RNase, purified using the QIAamp DNA Micro Kit (Qiagen), and sheared into fragments. Exon capture was performed with the SureSelect XT Mouse All Exon Kit (Agilent, Santa Clara, CA, USA). Exon capture libraries were sequenced using a paired-end protocol on the Illumina NovaSeq 6000 platform (Illumina, San Diego, CA, USA). The sequencing data were deposited in the BioProject database of the National Center for Biotechnology Information with the accession number PRJNA890699.

### 2.5. Exome Data Analysis

The sequencing reads were trimmed by removing low-quality bases and aligned to the mouse reference genome (GRCm38/mm10). Duplicate removal and base quality recalibration were performed using the Genome Analysis Toolkit (Broad Institute). The 20× mean depth coverage rate for the samples was 85.86 ± 1.12%. On average, 45.48 ± 3.01 M mapped reads with a duplication rate of 22.65 ± 1.65% were obtained. Structural variants were called using Manta v.1.6.0 [12] and were annotated using AnnotSV v2.2 [13]. For copy number variant analysis, segments were filtered for significance using the following criteria: Wilcoxon’s rank sum test with a *p*-value < 0.001, the Kolmogorov–Smirnov test with a *p*-value < 0.001, and uncertainty between 0 and 20. For insertion/deletion (indel) and single-nucleotide variant (SNV) detection, genetic variants were annotated with ANNOVAR [14], and the effect of each variant on the coding sequences was predicted using SnpEff v5.1 [15].

## 3. Results

### 3.1. Spontaneous Tumor Formation

**Figure 1:**
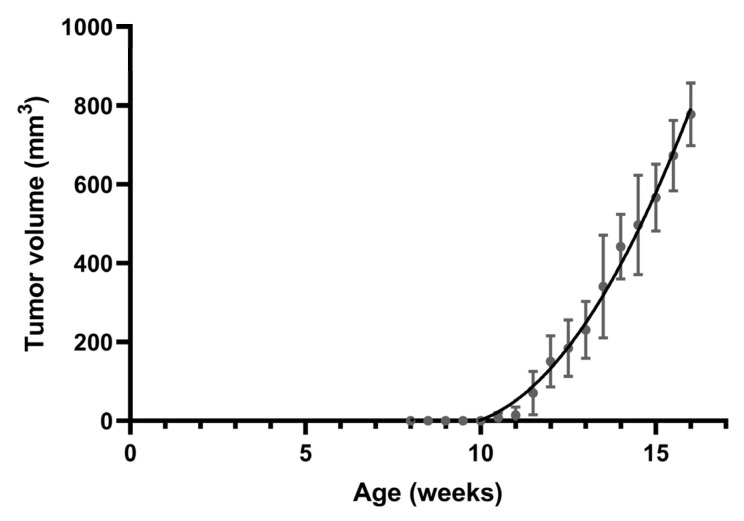
Spontaneous mammary tumor growth in female MMTV-PyVT mice. Data are shown as mean ± SD (*n* = 7).

All MMTV-PyVT mice develop spontaneous tumors arising from the mammary pads within a predictable time frame (Figure 1). Tumor formation was generally visible at an average of 11 weeks of age. We chose four time points corresponding to breast tumor formation for histology and whole-mount analysis: week 6 for hyperplasia, week 9 for ductal carcinoma in situ (DCIS), week 12 for early carcinoma, and week 16 for late carcinoma.

### 3.2. Histological Analysis

**Figure 2:**
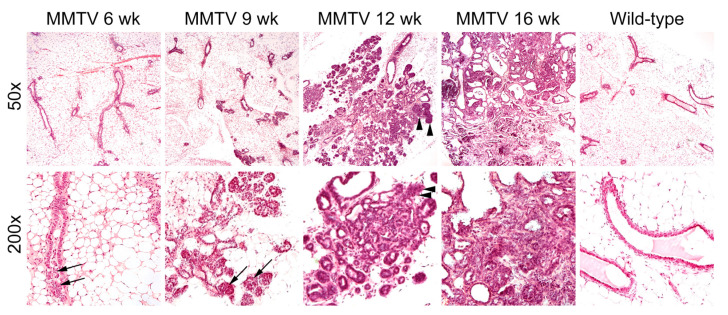
Hematoxylin and eosin staining of mammary glands in female wild-type and MMTV-PyVT transgenic mice. Original magnification: upper panel, ×50; lower panel, ×200. Arrows, hyperplastic intraductal cells; arrowheads, neoplastic growth through the basement membrane.

At 6 weeks of age, low-grade proliferation of the polarized glandular epithelial cells was evident on the H&E-stained sections of mammary glands (Figure 2). Most cells had a columnar shape with a low mitotic index. At 9 weeks, hyperplastic cells were haphazardly arranged along the duct wall of the terminal duct lobular unit. The cell borders were indistinct, and some neoplastic cells were loosely adhered to the duct walls. The neoplastic proliferation was confined within the lumens of the involved ducts and lobules. At 12 weeks of age, neoplastic cells with mild to moderate atypia breached the basement membrane around the lobular glands. Reactive alterations were present in the surrounding stroma. At 16 weeks, apparent features of late-stage carcinoma were accompanied by a grossly palpable lesion. The fibrotic tumor stroma was dense and collagenous, and angiolymphatic space invasion was sparsely identified.

**Figure 3:**
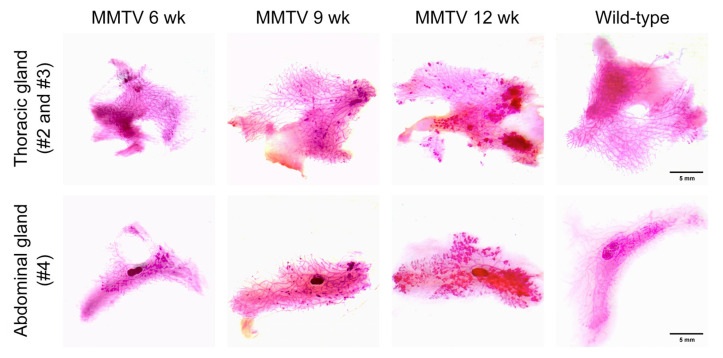
Carmine-alum-stained thoracic (second and third) and abdominal (fourth) mammary glands of female wild-type and MMTV-PyVT transgenic mice. Scale bar, 5 mm.

Thoracic and abdominal mammary fat pads containing entire mammary glands were placed on whole-mount slides and stained with carmine alum to depict the epithelial structures of the mammary gland. A higher branching density and increased staining intensity in the bulb-shaped terminal end buds were observed as early as 6 weeks of age (Figure 3). Along with tumor progression, there was an increase in the number and extent of hyperplastic areas.

### 3.3. Mouse-Level Genetic Alterations

We performed WES on mammary tumor and tail genomic DNA to identify structural variants and mutations in the MMTV-PyVT transgenic mice. Genetic variants in the WES data were identified using the mm10 mouse reference genome, and only variants present in all four samples were included. As shown in Table 1, the majority of variants were deletions, with the lengths of the variants ranging from 51 to 57,900 bp. Small indels are listed in Table 2. It is noteworthy that *Muc4*, an oncogene in tier-2 genes in the Cancer Gene Census, had frameshift indels on chromosome 16. SNVs with nonsynonymous mutations are listed in Table 3. We did not find nonsynonymous SNVs among the genes in the Cancer Gene Census [16].

### 3.4. Tumor-Level Genetic Alterations

The WES data from the mammary tumors were compared with those from matched tails. Genetic variants present in the mammary tumor DNA but not in the tail genomic DNA were considered somatic. There were no somatic structural alterations or copy number variations. Two oncogenes in tier-1 genes in the Cancer Gene Census, *Kat6a* and *Kmt2d*, had non-frameshift deletions. Table 4 lists somatic indels and SNVs of the mammary tumors from the MMTV-PyVT transgenic mice.

## 4. Discussion

Mouse models of breast cancer play an important role in the study of disease mechanisms and conduction of in vivo pharmacological testing. However, heterogeneity is quite common between models and within models [17]. Several large-scale analyses have been performed to compare the genetic perturbations of mammary lesions arising from different models or during the process of metastasis [3,5,6,17,18,19,20]. Discrepancies are the rule, possibly because multiple aberrations may be acquired early or late in breast tumorigenesis. Any attempt to clarify the pathogenesis may provide a new piece of the puzzle that will allow us to further understand the molecular basis of breast cancer initiation and progression.

Two of the principal signaling pathways that are stimulated by the PyVT are the mitogen-activated protein kinase (MAPK) and phosphatidylinositol 3-kinase (PI3K) cascades [21]. A few genetic alterations in this transgenic model have been reported previously. Src activation has been shown to play a pivotal role in PyVT-induced tumor formation [4,22]. Copy number alterations in key extracellular matrix proteins, including *Col1a1* and *Chad*, were shown to drive metastasis in MMTV-PyVT transgenic mice [19]. In another recent study, the presence of previously unreported recurrent mutations in *Shc1*, as well as recurrent oncogenic mutations in *Kras* and *Ctnnb1*, was a key factor driving metastasis in MMTV-PyVT mice [20]. As in real-world patients, the acquisition of varying aberrations occurs in different mice and in different laboratories.

In this study, we found that *Muc4* had frameshift indels in our MMTV-PyVT mice. MUC4 is a member of the transmembrane mucin family, and aberrant expression has been reported in a variety of carcinomas [23]. Aberrantly expressed MUC4 can act as a ligand for ERBB2, potentiate the phosphorylation of ERBB2, and reduce the binding of anti-ERBB2 antibodies to tumor cell surfaces [24]. Recently, it was demonstrated that Muc4 may facilitate tumor cell survival in circulation and, therefore, metastasis by promoting the association of circulating tumor cells with blood cells [25]. Pulmonary metastasis is commonly observed in MMTV-PyVT transgenic mice. In this regard, the frameshifts in *Muc4* may alter Muc4 expression and function and contribute to the high prevalence of metastasis. An important limitation of the current study is that we did not determine Muc4 expression during breast tumor development and progression. Moreover, it would be intriguing to correlate the expression level of Muc4 with proliferative indexes (such as Ki-67) or the expression of pro- and anti-apoptotic markers in breast tumors.

We demonstrated that mammary tumors arise in MMTV-PyVT mice through a multistage process, as in human breast cancer. However, while MMTV-PyVT transgenic mice are very useful in preclinical research, this genetically engineered mouse model does not recapitulate all aspects of human breast cancer. During the development of human breast cancer, gains in oncogene function or losses of tumor suppressor genes occur in a limited number of cells, whereas transgene effects are found throughout the mammary epithelial cells [26]. Nonetheless, MMTV-PyVT mice provide a versatile platform for studying various facets of breast tumorigenesis.

To summarize, we validated MMTV-PyVT transgenic mice as a multistage model for mammary carcinoma development and progression through histological analysis and whole-mount carmine alum staining. Constitutional and somatic genomic alterations were determined using WES, and possible pathogenic frameshift indels of *Muc4* were identified. Our characterization may be used as a reference for guidance in future research on MMTV-PyVT transgenic mice.

## Figures and Tables

**Table 1:** Structural variants of MMTV-PyVT transgenic mice.

Location	Gene	Accession No.	Class
Chr1:88234242-88234312	*Mroh2a*	NM_001281466	DEL, INV
Chr1:88266278-88266329	*6430706D22Rik*	NR_040291	DEL
Chr1:88270315-88271447	*A730008H23Rik*	NM_172505	DEL
Chr1:88270315-88271447	*Hjurp*	NM_198652	DEL
Chr1:171345007-171356531	*Nit1*	NM_012049	DEL, BND
Chr1:171356616-171356702	*Pfdn2*	NM_001360825	DEL
Chr6:116022400-116024804	*Tmcc1*	NM_001364577	DEL
Chr7:24082235-24085425	*Zfp180*	NM_001045486	DEL
Chr7:79651636-79665662	*Ticrr*	NM_029835	DEL
Chr7:84947480-84947640	*Vmn2r65*	NM_001105180	DEL
Chr7:141638851-141639059	*Muc6*	NM_001368953	DEL
Chr8:35482726-35491202	*Eri1*	NM_026067	DEL
Chr9:56260762-56318663	*Peak1*	NM_172924	DEL
Chr10:81178233-81178679	*Eef2*	NM_007907	DEL
Chr11:3137135-3137136	*Sfi1*	NM_030207	INS, BND
Chr12:106051004-106051759	*Vrk1*	NM_001029843	DEL
Chr19:21598332-21598752	*1110059E24Rik*	NM_025423	DEL

Abbreviation: DEL, deletion; INS, insertion; INV, inversion; BND, break-end.

**Table 2:** Insertion/deletion (Indel) mutations of MMTV-PyVT transgenic mice.

Location	Gene	Accession No.	Indel	Type
Chr1:139237087-139237087	*Crb1*	NM_133239	c.3481delC, p.R1161Gfs*48	frameshift deletion
Chr1:85094333-85094335	*A530032D15Rik*	NM_213615	c.392_394del, p.A131del	non-frameshift deletion
Chr1:85591567-85591567	*Sp110*	NM_030194	c.538dupG, p.A180Gfs*26	frameshift insertion
Chr1:88212372-88212372	*Ugt1a1*	NM_201645	c.371delT, p.M124Sfs*3	frameshift deletion
Chr1:88216161-88216162	*Ugt1a2*	NM_013701	c.1108_1109del, p.I370Hfs*10	frameshift deletion
Chr1:9546104-9546105	*Rrs1*	NM_021511	c.581_582del, p.H197Qfs*18	frameshift deletion
Chr3:130040795-130040797	*Sec24b*	NM_207209	c.751_753del, p.Q251del	non-frameshift deletion
Chr3:96683463-96683463	*Ankrd35*	NM_001081139	c.1065dupA, p.N356Kfs*9	frameshift insertion
Chr4:63171423-63171423	*Kif12*	NM_010616	c.179_180insGCCGGGTGGAGGCCC, p.P60_D61insPGGGP	non-frameshift insertion
Chr5:32737988-32737988	*Pisd*	NM_177298	c.C994T, p.R332X	stopgain
Chr5:93637618-93637618	*Pramel34*	NM_001164284	c.C802T, p.Q268X	stopgain
Chr8:104182034-104182034	*Bean1*	NM_001141922	c.42delC, p.Q15Kfs*29	frameshift deletion
Chr8:26160857-26160858	*Thap1*	NM_199042	c.154_155del, p.S52Hfs*12	frameshift deletion
Chr9:103355194-103355204	*Cdv3*	NM_175833	c.805_815del, p.V269Sfs*16	frameshift deletion
Chr9:39484333-39484333	*Or8g20*	NM_146830	c.919delA, p.I307*	stopgain
Chr9:65280131-65280131	*Cilp*	NM_173385	c.3507delG, p.G1170Afs*16	frameshift deletion
Chr13:64921972-64921972	*Spata31*	NM_030047	c.C1933T, p.R645X	stopgain
Chr16:32752550-32752550	*Muc4*	NM_080457	c.2427_2428insCA, p.Q810Hfs*30	frameshift insertion
Chr16:44496429-44496431	*Boc*	NM_172506	c.1348_1350del, p.S450del	non-frameshift deletion
Chr16:45729926-45729926	*Abhd10*	NM_001272070	c.870delC, p.D291Tfs*15	frameshift deletion
Chr17:23291424-23291424	*Vmn2r114*	NM_001102584	c.C2081G, p.S694X	stopgain
Chr17:23475353-23475353	*Vmn2r117*	NM_001104581	c.G1519T, p.G507X	stopgain

**Table 3:** Nonsynonymous single-nucleotide variants (SNVs) of MMTV-PyVT transgenic mice.

Location	Gene	Accession No.	SNV
Chr1:12839899-12839899	*Sulf1*	NM_172294	c.G1918A, p.D640N
Chr1:177773679-177773679	*Adss*	NM_007422	c.C686T, p.P229L
Chr1:26687400-26687400	*4931408C20Rik*	NM_001033764	c.T27A, p.N9K
Chr1:59847167-59847167	*Bmpr2*	NM_007561	c.G962A, p.R321Q
Chr1:75486756-75486756	*Obsl1*	NM_178884	c.C5291T, p.T1764M
Chr1:85246950-85246950	*C130026I21Rik*	NM_175219	c.C863T, p.S288L
Chr1:85615212-85615212	*Sp140*	NM_001013817	c.C443G, p.T148R
Chr2:131936461-131936461	*Prnp*	NM_011170	c.T32A, p.L11H
Chr2:84872044-84872044	*Rtn4rl2*	NM_199223	c.G1165C, p.A389P
Chr3:144691910-144691910	*Sh3glb1*	NM_019464	c.T980A, p.L327Q
Chr3:15548939-15548939	*Sirpb1b*	NM_001173460	c.A82G, p.M28V
Chr3:95734876-95734876	*Ecm1*	NM_007899	c.A1396G, p.I466V
Chr3:96854557-96854557	*Pdzk1*	NM_021517	c.A484G, p.N162D
Chr4:138221673-138221673	*Hp1bp3*	NM_010470	c.C46T, p.L16F
Chr4:140798123-140798123	*Padi3*	NM_011060	c.T578C, p.L193P
Chr4:147510785-147510785	*Zfp982*	NM_001039209	c.A63C, p.E21D
Chr4:147581328-147581328	*Zfp985*	NM_001014397	c.T117G, p.I39M
Chr4:147613775-147613775	*Zfp979*	NM_145078	c.C476T, p.T159I
Chr4:148944359-148944359	*Casz1*	NM_027195	c.T3260C, p.L1087P
Chr4:21873684-21873684	*Pnisr*	NM_025669	c.C1426G, p.R476G
Chr4:3184971-3184971	*Vmn1r3*	NM_001167535	c.C335T, p.T112I
Chr5:112762721-112762721	*Myo18b*	NM_028901	c.G5804A, p.R1935H
Chr5:114398443-114398443	*Ube3b*	NM_054093	c.T749G, p.M250R
Chr5:13570208-13570208	*Sema3a*	NM_009152	c.A1423G, p.I475V
Chr5:26035024-26035024	*Speer4a*	NM_029376	c.A727C, p.T243P
Chr5:27501274-27501274	*Speer4b*	NM_028561	c.C94T, p.P32S
Chr5:38300085-38300085	*Otop1*	NM_172709	c.G1187C, p.G396A
Chr5:89775351-89775351	*Adamts3*	NM_177872	c.G595A, p.V199I
Chr5:96758142-96758142	*Fras1*	NM_175473	c.T9404C, p.L3135P
Chr6:39400456-39400456	*Mkrn1*	NM_018810	c.A1036T, p.N346Y
Chr7:102973309-102973309	*Or51g2*	NM_147109	c.G682A, p.V228I
Chr7:105434593-105434593	*Cckbr*	NM_007627	c.C727G, p.R243G
Chr7:108465371-108465371	*Or5p73*	NM_146307	c.T46A, p.F16I
Chr7:120135179-120135179	*Zp2*	NM_011775	c.C1646T, p.A549V
Chr7:122167650-122167650	*Plk1*	NM_011121	c.C1090T, p.R364W
Chr7:131065072-131065072	*Dmbt1*	NM_007769	c.C1342A, p.P448T
Chr7:13801414-13801414	*Sult2a1*	NM_001111296	c.A713G, p.Q238R
Chr7:141858623-141858623	*Muc5b*	NM_028801	c.T5305C, p.Y1769H
Chr7:3222537-3222537	*Nlrp12*	NM_001033431	c.A3101G, p.K1034R
Chr7:43187290-43187290	*Zfp936*	NM_001034893	c.G124A, p.A42T
Chr7:56131292-56131292	*Herc2*	NM_010418	c.G3704A, p.G1235D
Chr7:79111354-79111354	*Acan*	NM_007424	c.A5813C, p.H1938P
Chr7:92858589-92858589	*Ddias*	NM_001080995	c.C2117T, p.P706L
Chr8:122890181-122890181	*Ankrd11*	NM_001081379	c.G6868C, p.V2290L
Chr9:109145537-109145537	*Fbxw21*	NM_177069	c.A914G, p.H305R
Chr9:120016907-120016907	*Xirp1*	NM_011724	c.A2909C, p.Q970P
Chr9:25130622-25130622	*Herpud2*	NM_020586	c.G253C, p.V85L
Chr9:38581513-38581513	*Or8b48*	NM_146810	c.C235T, p.P79S
Chr9:44249891-44249891	*Pdzd3*	NM_133226	c.T472C, p.C158R
Chr9:44942695-44942695	*Ube4a*	NM_145400	c.A1747T, p.N583Y
Chr10:58231344-58231344	*Dux*	NM_001081954	c.G1332C, p.L444F
Chr10:67238174-67238174	*Jmjd1c*	NM_001242396	c.T5144C, p.L1715P
Chr10:79169477-79169477	*Vmn2r80*	NM_001103368	c.A947G, p.N316S
Chr10:88091833-88091833	*Pmch*	NM_029971	c.T395C, p.I132T
Chr11:46222615-46222615	*Cyfip2*	NM_133769	c.C2903T, p.S968F
Chr11:90480671-90480671	*Stxbp4*	NM_011505	c.G1603A, p.A535T
Chr13:100161909-100161909	*Naip2*	NM_010872	c.T1618A, p.Y540N
Chr13:21468303-21468303	*Nkapl*	NM_025719	c.G139C, p.G47R
Chr13:27272475-27272475	*Prl3a1*	NM_025896	c.C311T, p.T104I
Chr13:53117204-53117204	*Ror2*	NM_013846	c.G1114A, p.V372M
Chr13:93063579-93063579	*Cmya5*	NM_023821	c.G10240C, p.A3414P
Chr14:51413192-51413192	*Vmn2r88*	NM_001368932	c.A361G, p.T121A
Chr14:70586204-70586204	*Fhip2b*	NM_194345	c.C1725A, p.S575R
Chr15:77638007-77638007	*Apol11b*	NM_001143686	c.T89G, p.I30R
Chr16:35291544-35291544	*Adcy5*	NM_001012765	c.G2770A, p.V924M
Chr16:36772445-36772445	*Slc15a2*	NM_021301	c.T629C, p.M210T
Chr16:38828345-38828345	*Tex55*	NM_029042	c.C401G, p.T134S
Chr16:39024953-39024953	*Igsf11*	NM_170599	c.G1045C, p.A349P
Chr16:43939116-43939116	*Ccdc191*	NM_027801	c.G1279A, p.V427I
Chr16:44299802-44299802	*Sidt1*	NM_198034	c.C515G, p.P172R
Chr16:44379308-44379308	*Spice1*	NM_144550	c.C2122A, p.R708S
Chr16:44789572-44789572	*Cd200r1*	NM_021325	c.A153G, p.I51M
Chr16:44820915-44820915	*Cd200r4*	NM_207244	c.T20C, p.I7T
Chr16:45094982-45094982	*Ccdc80*	NM_026439	c.A100G, p.T34A
Chr16:45239239-45239239	*Btla*	NM_177584	c.A305G, p.Q102R
Chr16:45392332-45392332	*Cd200*	NM_010818	c.A751G, p.I251V
Chr16:45539592-45539592	*Slc9c1*	NM_198106	c.T8C, p.M3T
Chr16:45664252-45664252	*Tmprss7*	NM_172455	c.G1544T, p.S515I
Chr16:46049747-46049747	*Cd96*	NM_032465	c.T1358C, p.F453S
Chr16:48817255-48817255	*Retnlb*	NM_023881	c.C43T, p.L15F
Chr17:35425194-35425194	*H2-Q6*	NM_207648	c.A151G, p.N51D
Chr17:35440154-35440154	*H2-Q7*	NM_010394	c.C580G, p.Q194E
Chr17:45517174-45517174	*Aars2*	NM_198608	c.C1810T, p.R604C
Chr17:47400410-47400410	*Guca1a*	NM_008189	c.A10G, p.I4V
Chr17:67752883-67752883	*Lama1*	NM_008480	c.G1966A, p.D656N
Chr18:24017781-24017781	*Zfp24*	NM_021559	c.A307T, p.I103F
ChrX:124127783-124127783	*Vmn2r121*	NM_001100616	c.A2539T, p.N847Y

**Table 4:** Somatic insertion/deletion and nonsynonymous single-nucleotide variants (SNVs) of mammary tumors arising from MMTV-PyVT transgenic mice.

Location	Gene	Accession No.	Amino Acid Change	Type
Chr3:15411378-15411378	*Sirpb1a*	NM_001002898	c.G559A, p.D187N	nonsynonymous SNV
Chr5:145803665-145803665	*Cyp3a44*	NM_177380	c.C164A, p.T55K	nonsynonymous SNV
Chr5:94535811-94535811	*Pramel42*	NM_001243937	c.T299A, p.L100H	nonsynonymous SNV
Chr6:29441097-29441102	*Flnc*	NM_001081185	c.1051_1054del, p.V351Pfs*16	frameshift deletion
Chr7:35409643-35409645	*Cep89*	NM_028120	c.546_548del, p.S190del	non-frameshift deletion
Chr8:122478985-122478987	*Ctu2*	NM_153775	c.546_548del, p.Q190del	non-frameshift deletion
Chr8:22935648-22935650	*Kat6a*	NM_001081149	c.3208_3210del, p.E1077del	non-frameshift deletion
Chr9:99583673-99583675	*Dbr1*	NM_031403	c.1303_1305del, p.E444del	non-frameshift deletion
Chr12:8728945-8728947	*Pum2*	NM_030723	c.1516_1518del, p.Q513del	non-frameshift deletion
Chr14:98168891-98168893	*Dach1*	NM_007826	c.417_419del, p.S156del	non-frameshift deletion
Chr15:101433138-101433138	*Krt87*	NM_001003668	c.T1226C, p.I409T	nonsynonymous SNV
Chr15:98846446-98846448	*Kmt2d*	NM_001033276	c.10830_10832del, p.Q3610del	non-frameshift deletion
Chr17:23348034-23348034	*Vmn2r115*	NM_001104579	c.G1519T, p.G507X	stopgain
Chr17:35873852-35873854	*Ppp1r18*	NM_175242	c.1678_1680del, p.E570del	non-frameshift deletion
Chr17:46412515-46412517	*Zfp318*	NM_207671	c.5443_5445del, p.E1823del	non-frameshift deletion

## Data Availability

The sequencing data of this study can be found in the BioProject database of the National Center for Biotechnology Information with the accession number PRJNA890699.
